# Periodontal Disease and Tooth Loss Are Associated with Lung Cancer Risk

**DOI:** 10.1155/2020/5107696

**Published:** 2020-07-27

**Authors:** You Chen, Bao-ling Zhu, Cong-cong Wu, Rui-fang Lin, Xi Zhang

**Affiliations:** ^1^College of Stomatology, Dalian Medical University, 9 West Section, Lvshun South Road, Lvshunkou District, Dalian City 116044, China; ^2^Department of Chemotherapy and Radiotherapy, The Second Affiliated Hospital and Yuying Children's Hospital of Wenzhou Medical University, 109 Xueyuan West Road, Lucheng District, Wenzhou City 325027, China

## Abstract

**Background:**

The associations between periodontal disease, tooth loss, and lung cancer risk remain debatable. Therefore, the purpose of the present study is to evaluate whether periodontal disease and tooth loss are associated with lung cancer risk.

**Methods:**

A literature search was performed for relevant studies using PubMed and Embase databases. Risk ratio (RR) with 95% confidence interval (CI) was applied as effect size to summarize the associations between periodontal disease, tooth loss, and lung cancer risk. A further dose-response analysis was also performed.

**Results:**

A total of twelve studies comprising 263,238 participants were included. The results indicated that periodontal disease was positively associated with lung cancer risk (RR = 1.37, 95%CI = 1.16‐1.63). There was a positive association between tooth loss and lung cancer risk (RR = 1.69, 95%CI = 1.46‐1.96). Moreover, there was a significantly linear dose-response relationship between tooth loss and lung cancer risk, and every 5 increment in tooth loss was associated with 10% increased lung cancer risk. Similar results were obtained in subgroup analysis.

**Conclusions:**

Periodontal disease and tooth loss are increased risk factors for lung cancer. Prevention and treatment of periodontal disease may be effective potential prevention strategies for lung cancer.

## 1. Introduction

Worldwide, lung cancer is the most frequently diagnosed cancer and the leading cause of cancer-related death, with approximately 2.1 million new lung cancer cases and 1.8 million deaths [1]. Surgical resection is the best radical treatment for lung cancer. However, most patients have lost the chance of radical resection at the initial diagnosis and are usually treated with palliative chemotherapy and/or radiotherapy. In spite of the improvement of the treatment level, the prognosis of lung cancer patients is still poor, with a low survival rate [2]. Therefore, early and effective prevention strategies for lung cancer risk are quite significant.

Periodontal disease is a chronic inflammatory disease of the periodontium caused by periodontal pathogen infection, resulting in the damage of tooth-supporting tissues and finally leading to tooth loss [3]. Severe periodontitis is the sixth-most prevalent public health condition worldwide, which has affected approximately 10.8% of the total population [4]. As a chronic inflammatory disease, several studies have reported that periodontal disease has been demonstrated to increase the risk of several malignancies such as head and neck cancer [5], oral cancer [6, 7], digestive tract cancer [8, 9], pancreatic cancer [10], and prostate cancer [11]. Unfortunately, there are no consistent conclusions on the associations between periodontal disease, tooth loss, and lung cancer risk because some studies show positive associations, while others show null associations.

Therefore, the purpose of our meta-analysis is to evaluate whether periodontal disease and tooth loss are associated with lung cancer risk and whether prevention of periodontal disease is an effective potential prevention strategy for lung cancer.

## 2. Material and Methods

### 2.1. Literature Search

A systematic literature search was performed for the relevant studies on associations between periodontal disease, tooth loss, and lung cancer risk using PubMed and Embase databases (up to September 2019). The search strategy was as follows: (periodontitis OR peridentitis OR “periodontal disease” OR “periodontal diseases” OR parodontopathy OR “gingival disease” OR “teeth number” OR “oral health” OR “dental health” OR “periodontal attachment loss” OR “periodontal pocket” OR “alveolar bone loss” OR gingivitis OR edentulous OR “tooth loss” OR “teeth loss” OR “dental plaque” OR edentulism) AND (lung cancer OR lung carcinoma OR lung tumor OR lung neoplasm). Furthermore, we manually searched the references of reviews and relevant studies to identify other eligible studies.

### 2.2. Eligibility Criteria

Included studies must meet the following eligibility criteria: (1) the exposure factor was periodontal disease and/or tooth loss; (2) the endpoint outcome was lung cancer risk; (3) the effect size of outcome was hazard ratio (HR), odds ratio (OR), or risk ratio (RR) with 95% confidence interval (CI); (4) the study type was cohort study or case-control study and the full text was available. In addition, only the most comprehensive study was included for meta-analysis if there were several duplicated studies. Some data reported only in the excluded duplicated studies were extracted into the included duplicated study for further analysis.

### 2.3. Data Extraction and Quality Assessment

Two reviewers independently screened literatures and extracted data as follows: first author, study type, study name, publication country and year, follow-up time, age, type of exposure ascertainment, type of lung cancer ascertainment, sample size, number of cases, exposure type, effect size with 95% CI, and controlled confounding factors. Moreover, if the same study provided several risk estimates and these risk estimates had major gaps for confounder control, only the risk estimates with the greatest control for confounding factors were extracted for meta-analysis. The study quality of the included studies was assessed by the Newcastle-Ottawa Scale (NOS) [12]. Any differences on literature selection, data extraction, and quality assessment were resolved by discussion.

### 2.4. Statistical Analysis

RR with 95% CI was used as effect size to summarize the associations between periodontal disease, tooth loss, and lung cancer risk. OR value provided by case-control study could be almost equal to the RR because the lung cancer incidence was extremely low, and thus, OR was used as RR in the data synthesis [13]. A pooled risk estimate was synthesized for further meta-analysis when there were multiple risk estimates based on different subpopulations in one study. For the meta-analysis of tooth loss, we utilized the risk estimate which represented the most severe degree of tooth loss since there was no uniform standard for tooth loss [10].

To assess the impact of the number of tooth loss on lung cancer risk, we also performed a dose-response analysis which needed the assigned values of tooth loss, distributions of cases and noncases, and risk estimates in each category [14]. A midpoint of the interval of the tooth loss was chosen as assigned value for the risk estimate in each category. For the open-ended interval [15], we assumed that the range was the same as that of the adjacent interval. Restricted cubic splines were utilized to test a nonlinear dose-response relationship, and generalized least-squares regressions were used to test a linear dose-response relationship [16, 17].

The Cochran *Q* test and the *I*^2^ statistic were performed to evaluate heterogeneity among studies, and the definition of statistically significant heterogeneity was *p* < 0.10 and/or *I*^2^ > 50% [18]. A random-effect model was used when the heterogeneity was significant, and on the contrary, a fixed-effect model was chosen. The Galbraith plot was used to explore which study contributed substantial heterogeneity. The overall analysis was performed by including all studies. Subgroup analysis was performed stratified by study type, study quality, cancer ascertainment, exposure ascertainment, sample size, and country. Moreover, adequate control of confounding factors was essential to obtain valid results and to reduce misleading results, and thus, subgroup analysis based on different controlled confounding factors was conducted. Moreover, we further performed in-depth subgroup analysis not only to control for smoking but also to control for smoking amount and duration because smoking was an extremely important risk factor for lung cancer [19]. Publication bias was evaluated by Egger's and Begg's tests [20, 21]. In addition, a trim-and-fill analysis was conducted to evaluate the impact of publication bias on the results when publication bias existed [22].

All statistical analyses were conducted in Stata software version 12.0 (Stata Corporation, USA). A two-sided *p* < 0.05 was considered statistically significant.

## 3. Results

### 3.1. Selection of Studies

**Figure 1:**
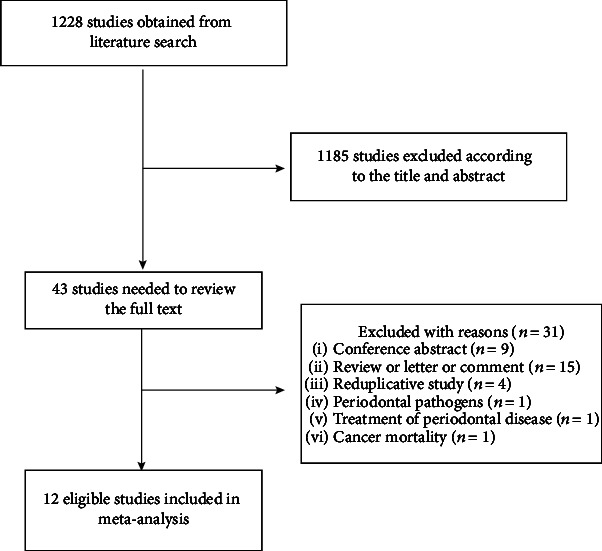
Process of literature search and study selection.

There were 1228 studies initially obtained from the literature search, among which 662 studies were from PubMed database and 566 studies were from Embase database. 1185 studies were excluded according to the title and abstract, and the remaining 43 studies were needed to review the full texts. After reviewing these full texts, 31 studies were excluded because these studies did not meet the eligibility criteria, and 12 studies were included for our meta-analysis [23–34]. The study selection process and the reasons for exclusion were shown in Figure 1.

### 3.2. Study Characteristics

The twelve studies were published from 2003 to 2019. Among these studies, five studies were from the USA, two studies were from Japan, and one study was from Turkey, Finland, Greece, Australia, and UK, respectively. In the type of study design, nine studies were cohort studies [24–28, 30, 31, 33, 34] and three studies were case-control studies [23, 29, 32]. In terms of exposure ascertainment, there were six studies using clinical periodontal examination [24, 26, 27, 29, 33, 34], and another six studies were self-reported measures [23, 25, 28, 30–32]. For the exposure factors, five studies only assessed periodontal disease [24, 27–30], three studies only assessed tooth loss [25, 32, 33], and four studies assessed both periodontal disease and tooth loss [23, 26, 31, 34]. For the control for confounding factors, age and sex were available in ten studies, smoking in eleven studies, alcohol drinking in eleven studies, BMI in seven studies, and diabetes in five studies. Moreover, among the included studies which have controlled for smoking, six and two studies further controlled for smoking amount and smoking duration, respectively. The main baseline characteristics of the included studies were shown in Table 1.

### 3.3. The Association between Periodontal Disease and Lung Cancer Risk

**Figure 2:**
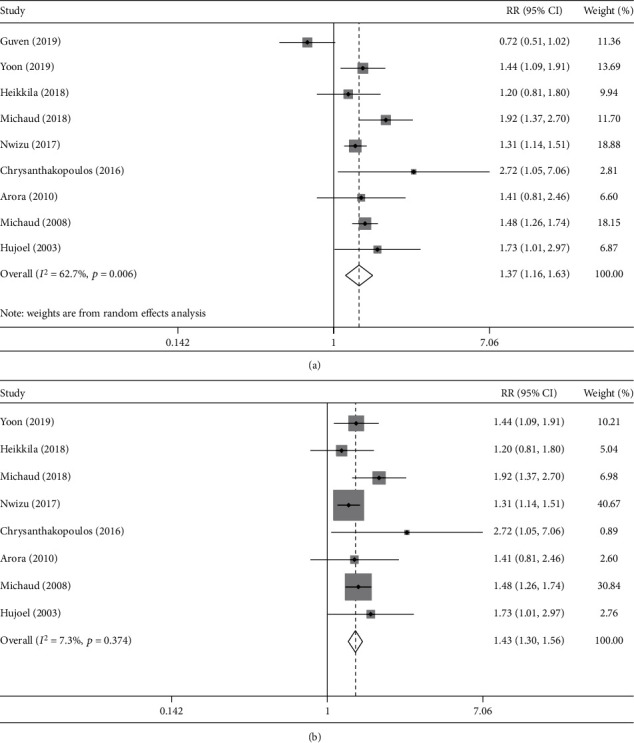
Result of the association between periodontal disease and lung cancer risk: (a) all studies; (b) excluding the study by Guven et al.

There were nine studies evaluating the association between periodontal disease and lung cancer risk [23, 24, 26–31, 34]. A random-effect model was used to pool RR due to a significant heterogeneity (*I*^2^ = 62.7%), and the result indicated a positive association between periodontal disease and lung cancer risk (RR = 1.37, 95% CI = 1.16‐1.63, Figure 2). The Galbraith plot showed that the study by Guven et al. contributed relatively substantial heterogeneity, and thus, we recalculated the pooled RR after excluding the study by Guven et al. [24]. The pooled RR with 95% CI indicated a consistent result, without significant heterogeneity (RR = 1.43, 95% CI = 1.30‐1.56, *I*^2^ = 7.3%, Figure 2). After excluding case-control studies, subgroup analysis based on cohort study also showed that periodontal disease could increase lung cancer risk (RR = 1.33, 95% CI = 1.09‐1.62). Subgroup analysis controlling for smoking indicated a positive relationship between periodontal disease and lung cancer risk (RR = 1.44, 95% CI = 1.31‐1.58), and similar results were also obtained after further controlling for smoking amount (RR = 1.40, 95% CI = 1.27‐1.54) and duration (RR = 1.86, 95% CI = 1.40‐2.48). The pooled RR with control for all potential important confounding factors (including age, sex, smoking, alcohol drinking, BMI, and diabetes) was 1.54 (95% CI = 1.34‐1.78), suggesting that periodontal disease was a strong factor for lung cancer risk (Table 2).

Moreover, similar results were acquired in the subgroup analysis after dividing into groups by gender, publication country, study quality, sample size, cancer ascertainment, exposure ascertainment, and various controlled confounding factors, indicating that the periodontal disease was positively associated with lung cancer risk (Table 2).

### 3.4. The Association between Tooth Loss and Lung Cancer Risk

**Figure 3:**
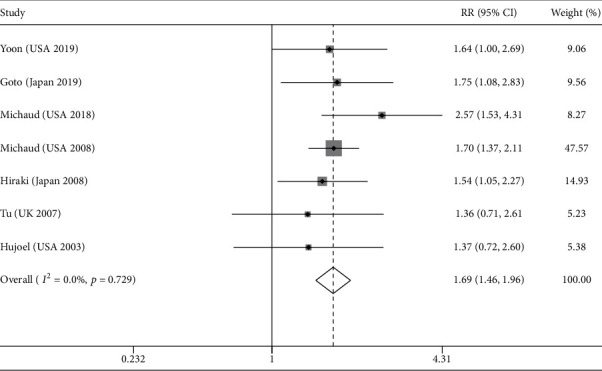
Result of the association between tooth loss and lung cancer risk.

There were seven studies estimating the association between tooth loss and lung cancer risk [23, 25, 26, 31–34]. The result indicated that there was a positive association between tooth loss and lung cancer risk (RR = 1.69, 95% CI = 1.46‐1.96, Figure 3), without significant heterogeneity (*I*^2^ = 0.0%). In terms of study design, subgroup analysis stratified by cohort study (RR = 1.73, 95% CI = 1.46‐2.05) and case-control study (RR = 1.58, 95% CI = 1.16–2.14) also showed a positive association. Subgroup analysis based on the control for smoking status, amount, and duration obtained similar results, and the pooled RR was 1.80 after controlling for all potential important confounding factors (including age, sex, smoking, alcohol drinking, BMI, and diabetes) (Table 2). Moreover, for the subgroup analysis after dividing into groups by publication country, study quality, gender, sample size, cancer ascertainment, exposure ascertainment, and various controlled confounding factors, we obtained similar results which indicated a positive relationship between tooth loss and lung cancer risk (Table 2).

**Figure 4:**
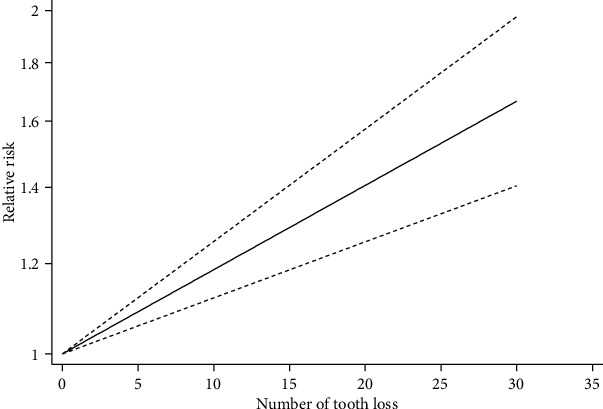
A linear dose-response relationship between tooth loss and lung cancer risk.

In addition, we performed a dose-response analysis to explore the impact of the number of tooth loss on lung cancer risk, and the result indicated that there was no nonlinear relationship (*p* for nonlinearity = 0.96). Indeed, a significant linear dose-response relationship was confirmed by the generalized least-squares regressions (*p* for linearity < 0.01, Figure 4). Every 5 increment in tooth loss was associated with 10% increased lung cancer risk (RR = 1.10, 95% CI = 1.04‐1.17).

## 4. Discussion

Lung cancer is the cancer of the highest morbidity and mortality worldwide which still have a poor prognosis even after effective treatment [1, 2]. Thus, it is urgent to find its risk factors for effective prevention. Recent studies have confirmed that periodontal disease and tooth loss are associated with several solid tumors such as oral cancer [6, 7], head and neck cancer [5], and pancreatic cancer [10]. However, the relationships between periodontal disease, tooth loss, and lung cancer risk are still controversial. Thus, we performed a meta-analysis to explore the relationships between periodontal disease, tooth loss, and lung cancer risk.

Twelve eligible studies comprising 263,238 participants were included in this meta-analysis. The results indicated that periodontal disease (RR = 1.37, 95% CI = 1.16‐1.63) and tooth loss (RR = 1.69, 95% CI = 1.46‐1.96) were positively associated with lung cancer risk. Moreover, similar results were obtained in subgroup analysis by study design, publication country, study quality, gender, sample size, cancer ascertainment, exposure ascertainment, and controlled confounding factors. The results of a dose-response analysis showed that there was a significantly linear relationship between tooth loss and lung cancer risk, and the lung cancer risk increased by 10% for 5 tooth increment in tooth loss, with a monotonically increasing trend.

In exploring the relationship between periodontal disease and lung cancer risk, four studies used self-reported measure to identify periodontal disease. Researchers may be concerned about whether self-reported periodontal disease was validated. Previous several systematic reviews have demonstrated that self-reported periodontal disease had acceptable validity and self-reported measure was feasible for monitoring periodontal disease in epidemiological studies [35, 36]. Moreover, numerous validation studies have also showed that self-reported measure was valid for the assessment of periodontal disease in different populations [37–40]. Indeed, the results of subgroup analysis based on self-reported periodontal disease showed that there was a positive relationship between periodontal disease and lung cancer risk (RR = 1.39, 95% CI = 1.26‐1.53), which was consistent with the results of subgroup analysis based on clinical periodontal examination. However, the adequacy for self-reported periodontal disease may depend on the education level and extent of access to routine oral healthcare in the population. Thus, the use of self-reported measure to identify periodontal disease may misclassify periodontal disease and underestimate the status of periodontal disease, which may weaken the association between periodontal disease and lung cancer risk. Future large-scale, well-designed diagnostic studies are needed to explore the validity of self-reported periodontal disease.

Periodontal disease and lung cancer were affected by many common risk factors [41, 42]. Therefore, the impact of these common confounding factors on the associations between periodontal disease, tooth loss, and lung cancer should be considered. We performed subgroup analyses based on various confounding factors, and the results confirmed the validity of our results. Among these controlled confounding factors, smoking was an extremely important risk factor for both periodontal disease and lung cancer. In order to extensively explore the impact of smoking on our results, it is essential not only to control for smoking but also to control for the smoking amount and duration, and our results still showed that there was a positive association between periodontal disease and lung cancer risk. Furthermore, a similar result was obtained after controlling for all potential important confounding factors (including age, sex, smoking, alcohol drinking, BMI, and diabetes), suggesting that periodontal disease was a strong risk factor for lung cancer risk. Future homogeneous, large-scale, and well-designed studies are needed to explore the associations between periodontal disease and lung cancer.

The mechanism of the positive relationship between periodontal disease and lung cancer was unclear. The associations between infection, inflammation, and lung cancer may be the most probable explanation [43]. Emerging evidence has indicated that infections could cause several types of malignant tumors, with approximately 1.2 million cases every year worldwide [44–46]. As a chronic inflammation caused by periodontal pathogen infections, periodontal disease could increase the levels of C-reactive protein, IL-6, IFN-*γ*, and IL-1*β* [47–49]. Periodontal pathogens and inflammation products entered into the bloodstream, which lead to systemic inflammatory response [50, 51]. Indeed, several studies have confirmed that high levels of C-reactive protein, IL-6, IFN-*γ*, and IL-1*β* were positively associated with lung cancer risk [52–54]. Dental plaque including supragingival plaque and subgingival plaque contained a good deal of bacteria in patients with periodontal disease [55]. Pneumonia caused by aspiration of oral bacteria may be another important mechanism [56–58]. Several studies have reported a positive relationship between pneumonia and lung cancer risk [59, 60]. Understandably, as a clinical indicator of periodontal disease, tooth loss could reflect the degree of poor oral health and was associated with lung cancer risk, with a linear relationship. Furthermore, further studies are required to explore the underlying mechanisms of the relationships between periodontal disease, tooth loss, and lung cancer risk.

Based on the present status that severe periodontitis and lung cancer were public health problems worldwide and there was a relationship between periodontal disease and lung cancer risk, it was a meaningful problem whether the risk of lung cancer could be reduced by effective prevention and treatment of periodontal disease [1, 4]. Some previous studies also have reported that the treatment of periodontal disease could reduce the level of inflammatory markers [61–64]. Moreover, Hwang et al. performed a retrospective cohort study including 116,706 periodontal disease patients to explore whether the treatment of periodontal disease could reduce the cancer risks [65]. The result indicated that the treatment of periodontal disease could reduce lung cancer risk in patients with periodontal disease after controlling for age, sex, occupation, type 2 diabetes mellitus, hypertension, and hyperlipidemia (RR = 0.45, 95% CI = 0.38‐0.54) [65]. However, this was an observational study and smoking was not controlled in the analysis, which may affect the validity of results and make it difficult to interpret the results. Therefore, there is an urgent need for large-scale, multicenter clinical studies to explore the prophylactic efficacy of treatment of periodontal disease for lung cancer risk after controlling for multiple confounding factors, especially smoking.

There was considerable heterogeneity in the meta-analysis of the association between periodontal disease and lung cancer risk. The result indicated that the study by Guven et al. contributed relatively substantial heterogeneity [24]. The reason may be that the number of lung cancer cases in the study was lower than that in other cohort studies due to a relatively short follow-up duration, and thus, the limited number of cases may affect the strength of the results. Moreover, the study only controlled for sex and age while the other studies also controlled for other important confounding factors as much as possible such as smoking and alcohol drinking. There was no significant heterogeneity in the meta-analysis of the association between tooth loss and lung cancer risk.

There were several limitations in our meta-analysis. First, the number of included studies was limited, which could affect the implementation of in-depth subgroup analyses. Second, the controlled confounding factors were varied, and the differences may be the potential source of heterogeneity. However, we could not completely control for all important confounding factors and eliminate the heterogeneity because the personal information could not be obtained from these published studies. Third, the exposure ascertainment of periodontal disease was discrepant. Some studies used self-reported measure to ascertain periodontal disease while the other studies used clinical periodontal examination to ascertain periodontal disease. Moreover, there was no uniform amount in the definition of tooth loss.

## 5. Conclusions

Our results indicate that periodontal disease and tooth loss are positively associated with lung cancer risk. Moreover, there is a significantly linear relationship between tooth loss and lung cancer risk, with a monotonically increasing trend. Moreover, subgroup analyses based on different controlled confounding factors including smoking status, amount, and duration also confirm the validity of our results. Further large-scale, well-designed studies are urgently required to adequately control for multiple confounding factors, especially smoking, to explore the association between periodontal disease, tooth loss, and lung cancer risk.

## Figures and Tables

**Table 1:** Baseline characteristics of the included studies.

Article	Country and year	Study design	Exposure measure	Cancer measure	Age (year)	Follow up (year)	Sample size	Case	Type of exposure	Adjusted variables	Study quality
Guven	Turkey 2019	Cohort study	Exam	Incidence	Median: 57.7	Median: 7.2	5199	32	Periodontal disease	Age and sex	5
Yoon	USA 2019	Case-control study	Self-reported	Incidence	40-79	NR	2015	403	Periodontal disease; tooth loss	BMI, education, household income, COPD, alcohol drinking, smoking status, pack-years, and missing data on oral health	6
Goto	Japan 2019	Cohort study	Self-reported	Mortality	35-70	Median: 10.2	11273	113	Remaining teeth	Age, sex, BMI, pack-years of smoking, alcohol consumption, education level, marital status, physical exercise, and medical history of hypertension and diabetes mellitus	7
Heikkila	Finland 2018	Cohort study	Exam	Mortality	Mean: 43	Mean: 10.1	68273	161	Periodontal disease	Calendar time, age, sex, socioeconomic status, number of teeth, dental treatments, oral health indices, need of periodontal treatment, and diabetes	7
Michaud	USA 2018	Cohort study	Exam	Incidence	44-66	Mean: 14.7	7466	226	Periodontal disease; edentulism	Age, field center, education level, smoking status, smoking duration, drinking status, BMI, diabetes status, sex, HRT use, and race	8
Nwizu	USA 2017	Cohort study	Self-reported	Incidence	54-86	Mean: 8.32 ± 3.95	65869	855	Periodontal disease	Age, pack-years, and BMI	5
Chrysanthakopoulos	Greece 2016	Case-control study	Exam	Incidence	Mean: 61.4 ± 4.2	NR	200	64	Periodontal disease	Gender, smoking, socioeconomic level, educational level, age, cancer family history, history of previous pulmonary disease, and annual dental follow-up	6
Arora	Australia 2010	Cohort study	Self-reported	Incidence	Median: 51 (range: 38-77)	Median: 27 (range: 1-41)	15333	225	Periodontal disease	Sex, age, education, employment, number of siblings, smoking status, smoking status of partner, alcohol status, diabetes, and BMI	5
Michaud	USA 2008	Cohort study	Self-reported	Incidence	Range: 40-75	Median: 17.7	48375	678	Periodontal disease; number of teeth	Age, ethnic origin, physical activity, history of diabetes, alcohol, BMI, geographical location, height, calcium intake, total calorific intake, red-meat intake, fruit and vegetable intake, vitamin D score, smoking history, and pack-years	5
Hiraki	Japan 2008	Case-control study	Self-reported	Incidence	58	NR	15720	909	Remaining teeth	Age, sex, smoking and drinking status, vegetable and fruit intake, BMI, and regular exercise	5
Tu	UK 2007	Cohort study	Exam	Mortality	Median: 19 (IQR: 3)	Mean: 46 ± 12	12223	NR	Tooth loss	Baseline smoking status	4
Hujoel	USA 2003	Cohort study	Exam	Mortality	Range: 25-74	About 17-21	11328	191	Periodontal disease; edentulism	Age, gender, poverty index, education, race, smoking duration and packs per day, cigar smoking, passive smoke, vitamins A & C and alcohol sampling design	7

BMI: body mass index; COPD: chronic obstructive pulmonary disease; HRT: hormone replacement therapy; IQR: interquartile range; NR: not reported; UK: United Kingdom; USA: United States of America.

**Table 2:** The results for the associations between periodontal disease, tooth loss, and lung cancer risk.

	*N*	RR	*p*_RR_	Heter	Publication bias
Periodontal disease					
Overall	9	1.37 (1.16-1.63)	<0.001	62.70%	Begg′s test = 0.602; Egger′s test = 0.771
Overall without Guven	8	1.43 (1.30-1.56)	<0.001	7.30%	Begg′s test = 0.386; Egger′s test = 0.168
Study type					
Cohort	7	1.33 (1.09-1.62)	0.004	68.80%	Begg′s test = 1.000; Egger′s test = 0.880
Cohort without Guven	6	1.42 (1.29-1.56)	<0.001	13.10%	Begg′s test = 0.707; Egger′s test = 0.460
Case-control study	2	1.52 (1.16-1.98)	0.002	37.00%	Begg′s test = 1.000; Egger's test = /
Cancer ascertainment					
Cancer incidence	7	1.37 (1.12-1.68)	0.002	70.40%	Begg′s test = 0.764; Egger′s test = 0.834
Cancer incidence without Guven	6	1.43 (1.30-1.57)	<0.001	21.00%	Begg′s test = 0.452; Egger′s test = 0.127
Cancer mortality	3	1.22 (1.02-1.45)	0.027	0.00%	Begg′s test = 0.296; Egger′s test = 0.374
Exposure ascertainment					
Exam	5	1.41 (0.90-2.21)	0.135	79.70%	Begg′s test = 0.806; Egger′s test = 0.493
Exam without Guven	4	1.65 (1.32-2.07)	<0.001	30.30%	Begg′s test = 1.000; Egger′s test = 0.637
Self-reported	4	1.39 (1.26-1.53)	<0.001	0.00%	Begg′s test = 1.000; Egger′s test = 0.719
Sample size					
<12000	5	1.46 (0.96-2.21)	0.075	79.70%	Begg′s test = 0.806; Egger′s test = 0.613
<12000 without Guven	4	1.67 (1.37-2.03)	<0.001	0.00%	Begg′s test = 0.308; Egger′s test = 0.278
≥12000	4	1.37 (1.24-1.51)	<0.001	0.00%	Begg′s test = 0.734; Egger′s test = 0.816
Country					
Not Asia	8	1.43 (1.30-1.56)	<0.001	7.30%	Begg′s test = 0.386; Egger′s test = 0.168
Sex					
Male	3	1.19 (0.65-2.17)	0.575	88.80%	Begg′s test = 1.000; Egger′s test = 0.717
Female	3	1.34 (1.17-1.52)	<0.001	0.00%	Begg′s test = 1.000; Egger′s test = 0.772
Study quality					
≥7	3	1.60 (1.27-2.03)	<0.001	37.2%	Begg′s test = 1.000; Egger′s test = 0.871
<7	6	1.30 (1.05-1.59)	0.014	69.1%	Begg′s test = 1.000; Egger′s test = 0.927
Adjusted variables					
Sex+age	8	1.37 (1.12-1.67)	0.002	67.10%	Begg′s test = 0.536; Egger′s test = 0.807
Smoking	7	1.44 (1.31-1.58)	<0.001	11.60%	Begg′s test = 0.368; Egger′s test = 0.072
Alcohol drinking	5	1.53 (1.36-1.73)	<0.001	0.00%	Begg′s test = 0.462; Egger′s test = 0.506
BMI	5	1.42 (1.30-1.56)	<0.001	12.3%	Begg′s test = 0.806; Egger′s test = 0.346
Diabetes	4	1.50 (1.31-1.71)	<0.001	9.3%	Begg′s test = 1.000; Egger′s test = 0.961
Smoking+alcohol drinking	5	1.53 (1.36-1.73)	<0.001	0.00%	Begg′s test = 0.462; Egger′s test = 0.506
Smoking+alcohol drinking+sex+age	4	1.55 (1.36-1.78)	<0.001	0.00%	Begg′s test = 1.000; Egger′s test = 0.530
Smoking+alcohol drinking+sex+age+BMI+diabetes	3	1.54 (1.34-1.78)	<0.001	0.0%	Begg′s test = 1.000; Egger′s test = 0.741
Adjusted smoking factor					
Amount of smoking	5	1.40 (1.27-1.54)	<0.001	0.0%	Begg′s test = 1.000; Egger′s test = 0.354
Duration of smoking	2	1.86 (1.40-2.48)	<0.001	0.0%	Begg′s test = 1.000; Egger's test = /
Tooth loss					
Overall	7	1.69 (1.46-1.96)	<0.001	0.00%	Begg′s test = 0.368; Egger′s test = 0.868
Study type					
Cohort	5	1.73 (1.46-2.05)	<0.001	0.00%	Begg′s test = 0.462; Egger′s test = 0.956
Case-control study	2	1.58 (1.16-2.14)	0.003	0.00%	Begg′s test = 1.000; Egger's test = /
Cancer ascertainment					
Cancer incidence	4	1.73 (1.47-2.05)	<0.001	0.00%	Begg′s test = 0.308; Egger′s test = 0.599
Cancer mortality	3	1.54 (1.10-2.14)	0.011	0.00%	Begg′s test = 0.296; Egger′s test = 0.011
Exposure ascertainment					
Exam	3	1.80 (1.28-2.54)	0.001	38.00%	Begg′s test = 0.296; Egger′s test = 0.028
Self-reported	4	1.67 (1.41-1.97)	<0.001	0.00%	Begg′s test = 0.734; Egger′s test = 0.679
Sample size					
<12000	4	1.82 (1.40-2.37)	<0.001	0.00%	Begg′s test = 0.734; Egger′s test = 0.605
≥12000	3	1.63 (1.36-1.96)	<0.001	0.00%	Begg′s test = 0.296; Egger′s test = 0.043
Country					
Asia	2	1.62 (1.20-2.19)	0.002	0.00%	Begg′s test = 1.000; Egger's test = /
Not Asia	5	1.72 (1.45-2.04)	<0.001	0.00%	Begg′s test = 0.221; Egger′s test = 0.902
Sex					
Male	3	1.66 (1.15-2.41)	0.007	70.40%	Begg′s test = 1.000; Egger′s test = 0.637
Female	2	1.49 (1.02-2.19)	0.040	0.00%	Begg′s test = 1.000; Egger's test = /
Study quality					
≥7	3	1.90 (1.39-2.58)	<0.001	17.2%	Begg′s test = 1.000; Egger′s test = 0.643
<7	4	1.64 (1.38-1.94)	<0.001	0.0%	Begg′s test = 0.308; Egger′s test = 0.140
Adjusted variables					
Sex+age	5	1.72 (1.47-2.02)	<0.001	0.00%	Begg′s test = 1.000; Egger′s test = 0.827
Smoking	7	1.69 (1.46-1.96)	<0.001	0.00%	Begg′s test = 0.368; Egger′s test = 0.868
Alcohol drinking	6	1.71 (1.47-2.00)	<0.001	0.00%	Begg′s test = 1.000; Egger′s test = 0.861
BMI	5	1.74 (1.48-2.03)	<0.001	0.0%	Begg′s test = 0.221; Egger′s test = 0.537
Diabetes	3	1.80 (1.50-2.16)	<0.001	4.7%	Begg′s test = 0.296; Egger′s test = 0.478
Smoking+alcohol drinking	6	1.71 (1.47-2.00)	<0.001	0.00%	Begg′s test = 1.000; Egger′s test = 0.861
Smoking+alcohol drinking+sex+age	5	1.72 (1.47-2.02)	<0.001	0.00%	Begg′s test = 1.000; Egger′s test = 0.827
Smoking+alcohol drinking+sex+age+BMI+diabetes	3	1.80 (1.50-2.16)	<0.001	4.7%	Begg′s test = 0.296; Egger′s test = 0.478
Adjusted smoking factor					
Amount of smoking	4	1.67 (1.40-1.99)	<0.001	0.0%	Begg′s test = 0.089; Egger′s test = 0.387
Duration of smoking	2	1.93 (1.05-3.57)	0.035	55.3%	Begg′s test = 1.000; Egger's test = /

Heter: heterogeneity; BMI: body mass index; *N*: the number of studies; RR: risk ratio; *p*_RR_: *p* value for the risk ratio; “/”: not applicable because Egger's test could not be conducted if the study number was only two.

## Data Availability

The data used to support the findings of this study are available from the corresponding author upon request.
